# Core/Whole Genome Multilocus Sequence Typing and Core Genome SNP-Based Typing of OXA-48-Producing *Klebsiella pneumoniae* Clinical Isolates From Spain

**DOI:** 10.3389/fmicb.2019.02961

**Published:** 2020-01-31

**Authors:** Elisenda Miro, John W. A. Rossen, Monika A. Chlebowicz, Dag Harmsen, Sylvain Brisse, Virginie Passet, Ferran Navarro, Alex W. Friedrich, S. García-Cobos

**Affiliations:** ^1^Department of Microbiology, Hospital de la Santa Creu i Sant Pau, Institut d’Investigació Biomèdica Sant Pau (IIB Sant Pau), Barcelona, Spain; ^2^Department of Medical Microbiology and Infection Prevention, University Medical Center Groningen, University of Groningen, Groningen, Netherlands; ^3^ESCMID Study Group for Genomic and Molecular Diagnostics (ESGMD), Basel, Switzerland; ^4^Department of Periodontology and Restorative Dentistry, University of Münster, Münster, Germany; ^5^Biodiversity and Epidemiology of Bacterial Pathogens, Institut Pasteur, Paris, France

**Keywords:** *K*. *pneumoniae*, OXA-48, cgMLST, wgMLST, WGS, molecular epidemiology

## Abstract

Whole-genome sequencing (WGS)-based typing methods have emerged as promising and highly discriminative epidemiological tools. In this study, we combined gene-by-gene allele calling and core genome single nucleotide polymorphism (cgSNP) approaches to investigate the genetic relatedness of a well-characterized collection of OXA-48*-*producing *Klebsiella pneumoniae* isolates. We included isolates from the predominant sequence type ST405 (*n* = 31) OXA-48-producing *K. pneumoniae* clone and isolates from ST101 (*n* = 3), ST14 (*n* = 1), ST17 (*n* = 1), and ST1233 (*n* = 1), obtained from eight Catalan hospitals. Core-genome multilocus sequence typing (cgMLST) schemes from Institut Pasteur’s BIGSdb-Kp (634 genes) and SeqSphere+ (2,365 genes), and a SeqSphere+ whole-genome MLST (wgMLST) scheme (4,891 genes) were used. Allele differences or allelic mismatches and the genetic distance, as the proportion of allele differences, were used to interpret the results from a gene-by-gene approach, whereas the number of SNPs was used for the cgSNP analysis. We observed between 0–10 and 0–14 allele differences among the predominant ST405 using cgMLST and wgMLST from SeqSphere+, respectively, and <2 allelic mismatches when using Institut Pasteur’s BIGSdb-Kp cgMLST scheme. For ST101, we observed 14 and 54 allele differences when using cgMLST and wgMLST SeqSphere+, respectively, and 2–5 allelic mismatches for BIGSdb-Kp cgMLST. A low genetic distance (<0.0035, a previously established threshold for epidemiological link) was generally in concordance with a low number of allele differences (<8) when using the SeqSphere+ cgMLST scheme. The cgSNP analysis showed 6–29 SNPs in isolates with identical allelic SeqSphere+ cgMLST profiles and 16–61 cgSNPs among ST405 isolates. Furthermore, comparison of WGS-based typing results with previously obtained MLST and pulsed-field gel electrophoresis (PFGE) data showed some differences, demonstrating the different molecular principles underlying these techniques. In conclusion, the use of the different WGS-based typing methods that were used to elucidate the genetic relatedness of clonal OXA-48-producing *K. pneumoniae* all led to the same conclusions. Furthermore, threshold parameters in WGS-based typing methods should be applied with caution and should be used in combination with clinical epidemiological data and population and species characteristics.

## Introduction

The accelerated spread of multidrug-resistant bacterial pathogens poses an important threat to public health (Zhao et al., 2010). One example is the marked increase of carbapenem-resistant *Klebsiella pneumoniae* producing different carbapenemases such as KPC, VIM, IMP, NDM, and OXA-48 (Oteo et al., 2014). Besides, the propensity of this species to cause outbreaks in healthcare institutions is well known (Snitkin et al., 2012). We need to understand the transmission of resistant pathogens to rapidly and effectively implement control measures during outbreak management (Zhou et al., 2016). Cluster identification and pathogen profiling, including antimicrobial resistance and virulence profile characterization, are crucial for effective infection control (Leopold et al., 2014; Zhou et al., 2015).

Pulsed-field gel electrophoresis (PFGE) and multilocus sequence typing (MLST) have been used as gold standards for many years to confirm suspected outbreaks. However, PFGE results are subjective and difficult to interpret (Leopold et al., 2014), and classical seven-gene MLST is an expensive and time-consuming method (Larsen et al., 2012). In contrast, whole-genome sequencing (WGS) is a technology with a proven high discriminatory power and has become more accessible due to recent technological advances (Larsen et al., 2012; Maiden et al., 2013; Bialek-Davenet et al., 2014; Leopold et al., 2014; Zhou et al., 2015, 2017; Rossen et al., 2018). However, defining criteria to interpret the data obtained by WGS in infection control is difficult and requires a comparison of available methods on well-characterized sets of isolates. Such criteria are advisable so that, following an outbreak, differences among isolates can be interpreted easily and accurately.

The objective of this work was to investigate the molecular epidemiology of a well-characterized collection of OXA-48*-*producing *K. pneumoniae* – the dissemination of which occurred in eight hospitals in Catalonia in 2012 (Argente et al., 2019) – using different WGS-based typing methods and to compare them with classical MLST and PFGE. We used the following WGS-based typing methods: (i) core-genome MLST (cgMLST), based on two different schemes, SeqSphere+ and BIGSdb-Kp (Snitkin et al., 2012); (ii) whole-genome MLST (wgMLST), based on the SeqSphere+ scheme (Zhou et al., 2016); and (iii) core-genome single nucleotide polymorphism (cgSNP) analysis. In addition, we investigated and compared phenotypic data and genotypic antimicrobial resistance data obtained using PCR and WGS.

## Materials and Methods

### Sample Collection

Thirty-seven OXA-48-producing *K*. *pneumoniae* isolates were selected from a previous study collection from 2012 and eight Catalan hospitals, including representative isolates of each MLST and PFGE profile, and antimicrobial resistance profile (Argente et al., 2019). These isolates were previously typed by PFGE (Argente et al., 2019) and MLST following the Institut Pasteur seven-gene MLST scheme^1^ (Diancourt et al., 2005). Information on phenotypic resistance and genotyping results from PCR and Sanger sequencing is summarized in Table 1. The selected isolates were from ST405 (*n* = 31, corresponding to E1–E18 PFGE patterns), ST101 (*n* = 3, PFGE patterns A1–A3), ST14 (*n* = 1, PFGE pattern D), ST17 (*n* = 1, PFGE pattern B), and ST1233 (*n* = 1, PFGE pattern C). The studied isolates did not belong to an outbreak, and neither had a known epidemiological link; thus, they were considered sporadic cases.

**TABLE 1:** Phenotypic and genotypic characters of the 37 OXA-48-producing *K*. *pneumoniae* strains obtained by conventional methods, PCR, and Sanger sequencing.

**MLST**	**PFGE**	**N° Strain**	**CEF**	**CXM**	**FOX**	**CTX**	**CAZ**	**ATM**	**FEP**	**ERT**	**IMP**	**Blc**	**K**	**T**	**G**	**A**	**N**	**Nm**	**AME**	**NAL**	**CIP**
101	A1	CARB115	**R**	**R**	**S**	**R**	**R**	**R**	**S**	**R**	**S**	*bla*_SHV–__1_	**R**	**R**	**R**	**S**	**S**	**R**	*aac(3′)*-IIa, *aac(6′)*-Ib	**R**	**R**
	A2	CARB077			R				R	R		*bla*_SHV–__1_						S	*aac(3′)*-IIa, *aac(6′)*-Ib		
	A3	CARB058			S				R	R		*bla*_SHV–__1_						R	*aac(3′)*-IIa, *aac(6′)*-Ib		

405	E1	CARB050	**R**	**R**	**R**	**R**	**R**	**R**	**I**	**R**	**S**	*bla*_SHV–__76_	**R**	**R**	**R**	**S**	**R**	**S**	*aac(3′)*-IIa	**R**	**R**
	E1	CARB009			S				R	I	S	*bla*_SHV–__76_				I			*aac(3′)*-IIa	I	
	E1	CARB007			S				S	I	S	*bla*_SHV–__76_	S	S		S	S		*aac(3′)*-IIa	S	S
	E1	CARB011			S				S	R	S	*bla*_SHV–__76_	S	S	S	S	S			I	S
	E1	CARB015			S		I		S	R	S	*bla*_SHV–__76_				S	S		*aac(3′)*-IIa	I	I
	E1	CARB037			S		S	I	S	I	S	*bla*_SHV–__76_	S	S		S	S		*aac(3′)*-IIa, *aac(6′)*-Ib	S	S
	E1	CARB125		I	S	S	S	S	S	I	S	*bla*_SHV–__76_			S	S	I		*aac(6′)*-Ib		
	E1	CARB102	I	S	S	S	S	S	S	I	S	*bla*_SHV–__76_			S	S	S		*aac(6′)*-Ib	S	I

	E2	CARB020	R	R	R	R	R	R	S	R	S	*bla*_SHV–__76_	R	R	R	S	S	S	*aac(3′)*-IIa, *aac(6′)*-Ib	R	R

	E3	CARB042	R	I	I	I	S	S	S	R	I	*bla*_SHV–__76_	R	R	R	S	S	S	*aac(6′)*-Ib	R	R

	E4	CARB106	R	R	S	R	R	R	I	I	S	*bla*_SHV–__76_	R	R	R	R	R	S	*aac(3′)*-IIa, *aac(6′)*-Ib	R	R

	E5	CARB182	**R**	**R**	**R**	**R**	**R**	**R**	**R**	**R**	**R**	*bla*_SHV–__76_	**R**	**R**	**R**	**R**	**R**	**S**	*aac(3′)-*IIa, *aac(6′)*-Ib	**R**	**R**
	E5	CARB056			I				S		S	*bla*_SHV–__76_				S	S		*aac(3′)-*IIa		
	E5	CARB022			S		S		S	S	S	*bla*_SHV–__76_				S	S		*aac(3′)-*IIa, *aac(6′)*-Ib	I	I
	E5	CARB128			S		S	S	S	S	S	*bla*_SHV–__76_	S	S		S	S		*aac(3′)-*IIa	S	S

	E6	CARB065	**R**	**R**	**R**	**R**	**R**	**R**	**R**	**R**	**I**	*bla*_SHV–__76_	**R**	**R**	**S**	**S**	**S**	**S**	*aac(3′)-*IIa, *aac(6′)*-Ib	**S**	**I**
	E6	CARB166	I	S	S	S	S	S	S		S	*bla*_SHV–__76_				I			*aac(6′)*-Ib	I	R

	E7	CARB038	R	R	R	R	R	R	I	R	S	*bla*_SHV–__76_	R	R	R	S	S	S	*aac(3′)-*IIa, *aac(6′)*-Ib	R	R

	E8	CARB100	R	R	S	R	R	R	S	R	S	*bla*_SHV–__76_	R	R	R	S	S	S	*aac(3′)-*IIa, *aac(6′′)*-Ib	I	I

	E9	CARB123	R	R	S	R	R	R	S	S	S	*bla*_SHV–__76_	R	R	R	S	S	S	*aac(3′)-*IIa, *aac(6′)*-Ib	S	I

	E10	CARB039	R	S	I	S	S	S	S	I	S	*bla*_SHV–__76_	R	R	R	S	R	S	*aac(6′)*-Ib	R	R

	E11	CARB184	R	R	S	R	R	R	S	S	S	*bla*_SHV–__76_	R	R	S	R	R	S	*aac(3′)-*IIa, *aac(6′)*-Ib	R	R

	E12	CARB112	**R**	**R**	**S**	**R**	**R**	**R**	**S**	**I**	**S**	*bla*_SHV–__76_	**R**	**R**	**R**	**S**	**S**	**S**	*aac(3′)-*IIa, *aac(6′)*-Ib	**R**	**R**
	E12	CARB130										*bla*_SHV–__76_					R		*aac(3′)-*IIa, *aac(6′)*-Ib	I	I

	E13	CARB096	R	R	S	R	R	R	S	R	S	*bla*_SHV–__76_	R	R	R	S	S	S	*aac(3′)-*IIa, *aac(6′)*-Ib	S	S

	E14	CARB010	R	R	S	R	I	R	S	R	S	*bla*_SHV–__76_	S	S	R	S	S	S	*aac(3′)-*IIa, *aac(6′)*-Ib	I	I

	E15	CARB026	R	R	S	R	R	R	S	R	S	*bla*_SHV–__76_	R	R	R	S	S	S	*aac(3′)-*IIa, *aac(6′)*-Ib	I	I

	E16	CARB183	R	R	S	R	R	R	I	I	S	*bla*_SHV–__76_	R	R	S	S	R	S	*aac(3′)-*IIa, *aac(6′)*-Ib	R	R

	E17	CARB044	S	S	S	S	S	S	S	S	S	*bla*_SHV–__76_	R	R	S	I	I	S	*aac(3′)-*IIa, *aac(6′)*-Ib	S	S

	E18	CARB040	**R**	**R**	**S**	**R**	**R**	**R**	**S**	**R**	**S**	*bla*_SHV–__76_	**R**	**R**	**R**	**S**	**I**	**S**	*aac(3′)-*IIa, *aac(6′)*-Ib	**R**	**R**
	E18	CARB139								S		*bla*_SHV–__76_					S	R	*aac(3′)-*IIa, *aac(6′)*-Ib	S	S
14	D	CARB117	R	S	S	S	S	S	S	I	S	*bla*_SHV–__1_	S	S	S	S	S	S		S	S
17	B	CARB098	I	S	S	S	S	S	S	S	S	*bla*_SHV–__11_	S	S	S	S	S	S		S	S
1233	C	CARB122	S	S	S	S	S	S	S	S	S	*bla*_SHV–__42_	S	S	S	S	S	S		S	S

*In bold, consensus sequence. Blank spaces regarding resistance phenotype indicate same result as consensus sequence, and regarding resistance genes (AME) indicate absence. Blc, beta-lactamase; AME, aminoglycoside-modifying enzymes. R, resistant; I, intermediate; S, susceptible; CEF, cephalotin; CXM, cefuroxime; FOX, cefoxitin; CTX, cefataxime; CAZ, ceftazidime; ATM, aztreonam; FEP, cefepime; ERT, ertapenem; IMP, imipenem; K, kanamycin; T, tobramycin; G, gentamicin; A, amikacin; N, netilmicin; Nm, neomycin; NAL, nalidixic acid; and CIP, ciprofloxacin; MLST, multilocus sequence type; PFGE, pulsed-field gel electrophoresis. All strains were resistant to ampicillin, piperacillin, coamoxiclav [except: CARB128 (E184) and CARB184 (E11)], and piperacillin/tazobactam [except: CARB122 (C), CARB128 (E5), and CARB184 (E11)]. All strains carried OXA-1 [except: CARB117 (D) and CARB122 (C)], TEM-1 [except: CARB042 (E3), CARB166 (E6), CARB117 (D), CARB098 (B), and CARB122 (C)], CTX-M-15 [except: CARB125 (E1), CARB102 (E1), CARB042 (E3), CARB039 (E10), CARB044 (E17), CARB117 (D), CARB098 (B), and CARB122 (C)], and QnrB [except: CARB077 (A2), CARB117 (D), CARB098 (B), and CARB122 (C)]. All strains had the Tn1999.2 genetic platform of OXA-48, except: CARB106 (E4), CARB166 (E6) CARB184 (E11) CARB112, and CARB130 (E12), and CARB183 (E16).*

### WGS

After DNA extraction using the Ultraclean Microbial DNA Isolation Kit (MO BIO Laboratories, Carlsbad, CA, United States), WGS was applied to the 37 OXA-48-producing *K*. *pneumoniae* isolates. The libraries were prepared using a Nextera XT v.01 kit (Illumina Inc., San Diego, CA, United States) and sequenced on an Illumina MiSeq sequencer (Illumina Inc., San Diego, CA, United States) to generate 2 × 250 bp paired-end reads. After sequencing, the reads were quality trimmed and *de novo* assembled using the CLC Genomics Workbench software v7.0.4 (CLC Bio, Aarhus, Denmark), with the default parameters, except for “removal of low quality sequence limit,” which was set to 0.01 to increase the quality of the called bases.

Assembly quality was assessed using QUAST v4.5 (Gurevich et al., 2013), and a report including assembly metrics was generated (Supplementary Table S1).

### Core-Genome and Whole-Genome MLST (cgMLST, wgMLST), Ridom SeqSphere+ Schemes

The assembled genomes were imported into SeqSphere+ software v5.1.0 (Ridom GmbH, Münster, Germany) for a gene-by-gene allele calling comparison using a higher number of loci than the already published Institut Pasteur’s BIGSdb-Kp cgMLST scheme (Bialek-Davenet et al., 2014). A hard defined and stable scheme comprising 2,365 targets or genes of the *K. pneumoniae* core genome (cgMLST) and 2,526 genes of the *K. pneumoniae* accessory genome (wgMLST; total of 4,891 targets), available in SeqSphere+, was used for the analysis (targets included in the schemes can be found at cgMLST.org). This scheme was developed using the seed genome *K. pneumoniae* subsp. *pneumoniae* NTUH-K2044 (GenBank accession no. NC_012731.1; 15-JUN-2016) and comparing it with 30 query genome sequences that cover the genetic variability of the whole species complex, i.e., *K. pneumoniae*, *K. variicola*, and *K. quasipneumoniae*, as previously defined (Bialek-Davenet et al., 2014). In addition, 61 plasmid sequences were used to exclude chromosomal homologs of plasmid genes. A detailed description of the scheme development is available in the software. In addition, a cgMLST complex type (CT) function was used to identify isolates with very similar cgMLST profiles^2^. A cluster alert distance of 15 cgMLST allele differences and a cluster alert quality threshold of at least 90% good cgMLST targets were used to detect closely related isolates. These thresholds were based on retrospective analysis of well-defined outbreaks and out-group isolates with the same MLST/MLVA/PFGE profiles. Distance matrices describing pairwise allele differences (ignoring missing values) are included in Supplementary Data Sheet S1 (cgMLST and wgMLST, SeqSphere+).

Furthermore, another parameter named the genetic distance was calculated for paired isolates as the proportion of allele differences, obtained by dividing the number of allele differences between the two genomes by the total number of called genes shared by those two genomes. We considered previously described genetic distance thresholds, 0.0035 for cgMLST and 0.0045 for wgMLST, to discriminate between epidemiologically related and unrelated *K. pneumoniae* isolates (Kluytmans-van den Bergh et al., 2016).

### cgMLST, BIGSdb-Kp Scheme

Assembled genomes were analyzed using the Institut Pasteur BIGSdb-Kp^3^ 634-gene strict core-genome MLST (scgMLST) scheme (Bialek-Davenet et al., 2014). A spanning tree (BioNumerics v6.7.3, Applied-Maths, Belgium) was constructed from the allelic profiles of this cgMLST scheme to visualize the corresponding diversity of genotypes (figure in Supplementary Figure S1).

### Core-Genome SNP Analysis

The number of single nucleotide polymorphisms (SNPs) in the core genome (defined as orthologous sequences conserve in all aligned genomes) was calculated from the *de novo* assembled genomes for isolates with the same ST (ST405 and ST101) using the Harvest software suite (parsnp), including recombination filtration (Treangen et al., 2014). A cgSNP-based tree was built using the Neighbor Joining method.

### Antimicrobial Resistance Genes and Plasmid Analysis

In addition to WGS typing, resistance genotypes and plasmid families were investigated in the collection of OXA-48-producing *K. pneumoniae* and compared with those obtained previously (Argente et al., 2019). Genome assemblies were uploaded to the Center for Genomic Epidemiology to extract information on antimicrobial resistance genes (ARGs) and plasmid replicons for *K. pneumoniae* using ResFinder 2.1 and PlasmidFinder 1.3, respectively (Zankari et al., 2012; Carattoli et al., 2014).

The *de novo* assembled genomes were annotated using Prokka 1.12 (Seemann, 2014) and compared with reference *K. pneumoniae* plasmid pOXA-48 NC_019154.1 using BLASTn analysis. Circular representation of compared genomes was performed with the Circular Genome Viewer (CGView) Comparison Tool program (Grant et al., 2012), in which the coding sequences (CDS) in reference and query sequences were used. In addition, generated blastn output files were further used for visualization using DNAplotter (Carver et al., 2009) and for further analysis using the Artemis Comparison Tool (Carver et al., 2005). Similarity matches were filtered based on their lengths (100 kb segments) and percentage similarity scores, and only the filtered hits with at least 80% sequence similarity were subsequently displayed using the Artemis Comparison Tool.

## Results

### cgMLST and wgMLST Schemes From Ridom SeqSphere+

**FIGURE 1:**
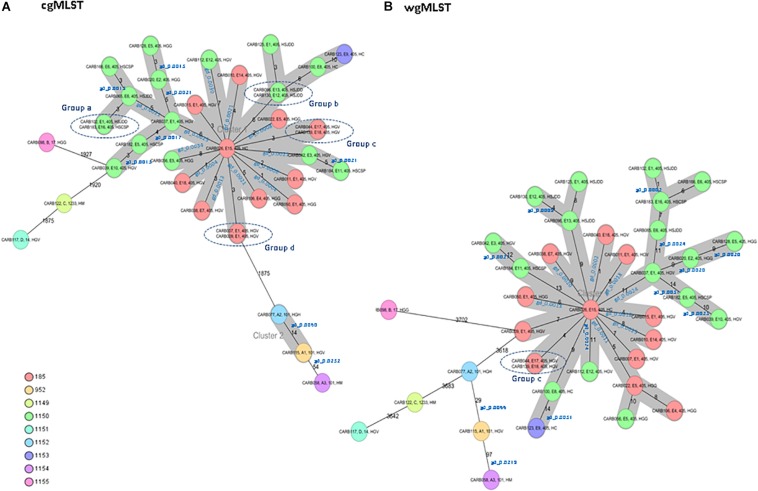
Minimum spanning tree of 37 OXA-48-producing *K. pneumoniae* isolates obtained from a previous study (Argente et al., 2019). Each circle represents a genotype, and the number of different alleles is indicated on the edges between connected isolates (nodes). In addition, the genetic distance (gd) for specific pairwise comparisons is indicated in blue (see also Table 2). Isolates are presented by their ID, pulsed-field gel electrophoresis (PFGE) profile, sequence type (ST), and hospital origin. Colors indicate the different complex types (CTs). The cluster distance threshold is 15. **(A)** Distance based on whole-genome multilocus sequence typing (cgMLST) of 2,365 genes using the parameters “pairwise ignoring missing values” during calculation. Groups of isolates (groups a, b, c, and d) with 0 allele differences obtained by cgMLST analysis are highlighted. **(B)** Distance based on whole-genome multilocus sequence typing (wgMLST) of 4,891 genes (cgMLST of 2,365 core genes and 2,526 accessory genes) using the parameters “pairwise ignoring missing values” during calculation. Isolates of group c that were previously investigated by cgMLST analysis are highlighted.

The cgMLST analysis of the 37 isolates resulted in nine CTs: 185, 952, and 1149 to 1155 (Figure 1A). Among isolates of MLST type ST405 (*n* = 31), three different CTs were assigned: CT185 (PFGE subtypes E1, E4, E5, E7, E14, E15, E17, and E18), CT1150 (PFGE subtypes E1, E2, E3, E5, E6, E8, E10–E13, and E16), and CT1153 (PFGE subtype E9). The three ST101 isolates (PFGE subtypes A1–A3) had different CTs (CTs 952, 1152, and 1154) (Figure 1A). ST14, ST17, and ST1233 isolates (*n* = 1, each ST) (PFGE profiles D, B, and C, respectively) were assigned to CTs 1151, 1155, and 1149, respectively. These minority STs (ST14, ST17, and ST1233) were clearly separated from the ST405 and ST101 isolates, with an allele distance from ST405 isolates of more than 1,875 allele differences when using cgMLST (Figure 1A), and more than 3,618 alleles when using wgMLST (Figure 1B).

Isolates within ST101, CT952 (PFGE-A1), and CT1152 (PFGE-A2) showed 14 different alleles by cgMLST (Figure 1A and Table 2) and differed in two bands by PFGE (97% similarity; data not shown). However, these isolates had a genetic distance of 0.0060 indicating no genetic relatedness. Clinical epidemiological data of isolates clustering together by cgMLST (<15 allele differences) are described in Table 3.

**TABLE 2:** Genetic distance for pairwise comparisons of OXA-48- *K. pneumoniae* isolates using cgMLST/wgMLST schemes from Ridom SeqSphere+ and cgMLST from BIGSdb-Kp (Institut Pasteur).

							**Ridom SeqSphere+ Schemes**				**BIGSdb-Kp Scheme**	
**Sample**	**Hospital**	**Isolation date in 2012 (month–day)**	**PFGE profile**	**ST**	**CT**^*a*^	***Minimum spanning tree results***	**No. alleles differences/no. shared genes cgMLST**^*b*^	**No. alleles differences/no. shared genes wgMLST**^*c*^	**Genetic distance cgMLST**^*d*^	**Genetic distance wgMLST**^*e*^	**No. alleles differences/no. shared genes cgMLST**^*f*^	**Genetic distance cgMLST**^*f*^
^1^CARB115	HGV	July-31	A1	101	952							
CARB077	HGH	May-05	A2	101	1152		14/2322	29/4416	*0.0060*	*0.0066*	2/623	0.0032
CARB058	HM	July-11	A3	101	1154		54/2328	97/4432	*0.0232*	*0.0219*	5/624	0.0080

CARB098	HGG	September-10	B	17	1155							

CARB122	HM	January-19	C	1233	952							

CARB117	HGV	October-18	D	14	1151							

CARB102	HSJDD	October-05	E1	405	1150	Group a	0/2332	1/4458	NA	0.0002	0/623	NA
CARB183	HSCSP	August-21	E16	405	1150	Group a						

CARB130	HSJDD	December-05	E12	405	1150	Group b	0/2334	4/4427	NA	0.0009	0/628	NA
CARB096	HSJDD	September-06	E13	405	1150	Group b						

CARB044	HGV	February-19	E17	405	185	Group c	0/2358	0/4504	NA	NA	0/629	NA
CARB139	HGV	December-29	E18	405	185	Group c						

CARB007	HGV	January-04	E1	405	185	Group d	0/2306	7/4388	0.0016	NA	1/623	0.0016
CARB009	HGV	January-19	E1	405	185	Group d						

^2^CARB026	HC	January-03	E15	405	185							
CARB050	HGG	June-20	E1	405	185		1/2351	6/4488	0.0004	0.0013	0/631	NA
CARB040	HGV	February-16	E18	405	185		1/2357	1/4505	0.0004	0.0002	0/630	NA
CARB011	HGV	January-15	E1	405	185		2/2354	8/4480	0.0008	0.0018	0/629	NA
CARB022	HGG	Mars-21	E5	405	185		2/2357	5/4501	0.0008	0.0011	0/630	NA
CARB038	HGV	May-10	E7	405	185		3/2356	9/4496	0.0013	0.0020	1/630	0.0016
CARB015	HGV	September-04	E1	405	185		3/2357	4/4500	0.0013	0.0009	1/630	0.0016
CARB010	HGV	January-11	E14	405	185		4/2357	8/4497	0.0017	0.0018	0/630	NA
CARB106	HGG	October-29	E4	405	185		5/2351	11/4474	0.0021	0.0024	0/628	NA
CARB042	HGV	April-04	E3	405	1150		5/2357	15/4499	0.0021	0.0033	1/629	0.0016
CARB037	HGV	May-14	E1	405	185		6/2355	11/4493	0.0025	0.0024	0/631	NA
CARB112	HGV	June-21	E12	405	1150		7/2356	11/4500	0.0030	0.0024	1/631	0.0016
CARB056	HGG	July-03	E5	405	1150		8/2349	13/4464	0.0034	0.0029	2/630	0.0032

CARB184	HSCSP	October-02	E11	405	1150							
CARB042	HGV	April-04	E3	405	1150		5/2358	12/4500	0.0021	0.0027	1/629	0.0016

CARB100	HC	October-05	E8	405	1150							
CARB123	HC	September-12	E9	405	1153		10/2357	14/4503	0.0042	0.0031	1/628	0.0031

^3^CARB037	HGV	May-14	E1	405	1150							
CARB065	HSJDD	August-19	E6	405	1150		5/2357	11/4493	0.0021	0.0024	0/628	NA
CARB182	HSCSP	July-19	E5	405	1150		4/2356	14/4492	0.0017	0.0031	0/631	NA
CARB020	HGG	Mars-06	E2	405	1150		5/2326	9/4419	0.0021	0.0020	1/624	0.0016

CARB020	HGG	Mars-06	E2	405	1150							
CARB128	HGG	Desember-10	E5	405	1150		3/2317	9/4397	0.0013	0.0020	1/623	0.0016

CARB182	HSCSP	July-19	E5	405	1150							
CARB039	HGV	May-08	E10	405	1150		3/2313	10/4411	0.0013	0.0023	0/630	NA

CARB065	HSJDD	August-19	E6	405	1150							
CARB166	HSCSP	September-19	E6	405	1150		3/2355	7/4489	0.0013	0.0015	0/627	NA

**^*a*^CT, complex type from cgMLST Ridom SeqSphere+. ^*b*^cgMLST = 2,365 targets including 7 MLST genes (Ridom SeqSphere+). ^*c*^wgMLST = 2,365 targets of cgMLST + 2329 accessory targets, 4,891 targets in total.*^*d*^Threshold = 0.0035 (Kluytmans-van den Bergh et al., 2016) (italic indicates genetic distance above the threshold). *^*e*^Threshold = 0.0045 (Kluytmans-van den Bergh et al., 2016) (italic indicates genetic distance above the threshold). ^*f*^cgMLST = 634 genes corresponding to the strict core genome (scgMLST634) scheme of Bialek-Davenet et al. (2014) (28) (described at bigsdb.pasteur.fr/klebsiella). ^1^CARB115 isolate used for pairwise comparison with CARB077 and CARB058. ^2^CARB026 isolate used for pairwise comparison with CARB050, CARB040, CARB011, CARB022, CARB038, CARB015, CARB010, CARB106, CARB042, CARB037, CARB112, and CARB056. ^3^CARB037 isolate used for pairwise comparison with CARB065, CARB182, and CARB020.cgMLST, core-genome multilocus sequence typing; wgMLST*, *whole-genome multilocus sequence typing; PFGE, pulsed-field gel electrophoresis; HSJDD, Hospital de Sant Joan de Déu (Manresa); HSCSP, Hospital de la Santa Creu i Sant Pau (Barcelona); HGV, Hospital General de Vic (Vic); HGH, Hopsital General de l’Hospitalet (Hospitalet del Llobregat); HM, Hospital de Mataró (Mataró).**

**TABLE 3:** Epidemiological and clinical context of OXA-48-producing *K*. *pneumoniae* isolates that clustered together using cgMLST.

**MLST**	**PFGE**	**WGS groups**	**Isolates**	**Hospital**	**Department**	**Isolation date**	**Clinical sample**	**Baseline pathology**	**Gender**	**Age**
ST405	E1	a	CARB102	HSJdD	Internal Medicine	10-05-12	Urine	Trasplanted	Woman	71
	E16		CARB183	HSCSP	Oncology	08-21-12	Urine	Leukemia	Man	60

	E12	b	CARB130	HSJdD	Cardiology	12-05-12	Wound	Cardiac dyspnea	Woman	98
	E13		CARB096	HSJdD	Dialysis Unit	09-06-12	Urine	Dialysis	Man	59

	E17	c	CARB044	HGV	Emergencies	02-19-12	Urine	UTI	Man	88
	E18		CARB139	HGV	Emergencies	12-28-12	Urine	UTI	Man	68

	E1	d	CARB007	HGV	Emergencies	01-04-12	Urine	UTI	Woman	76
	E1		CARB009	HGV	Emergencies	01-19-12	Urine	UTI	Woman	91

ST101	A2		CARB077	HGH	Emergencies	05-11-12	Rectal exudate	ESBL-carrier	Man	82
	A1		CARB115	HGV	Emergencies	08-25-12	Urine	UTI	Woman	89
	A3		CARB058	HM	Emergencies	07-11-12	Urine	UTI	Woman	87

**MLST, multilocus sequence typing; PFGE, pulsed-field gel electrophoresis; WGS, Whole-genome sequencing; UTI, urinary tract infection; ESBL, extended-spectrum beta-lactamase; HSJdD, Hospital de Sant Joan de Déu (Manresa); HSCSP, Hospital de la Santa Creu i Sant Pau (Barcelona); HGV, Hospital General de Vic (Vic); HGH, Hopsital General de l’Hospitalet (Hospitalet del Llobregat); HM, Hospital de Mataró (Mataró).**

Isolates within ST405 (PFGE subtypes E1–E18, which differed in less than three bands between them) differed by a maximum of 10 alleles by cgMLST and 14 alleles by wgMLST (Figures 1A,B). All of them had a genetic distance below the threshold (0.0035) and belonged to CT185 or CT1150, except isolates CARB100 (CT1150) and CARB123 (CT1153), which showed a genetic distance of 0.0042 (10 allele differences) by cgMLST (Table 2). In contrast, when considering the wgMLST analysis, CARB100 and CARB123 had a genetic distance of 0.0031 (14 allele differences) indicating genetic relatedness (Table 2). By PFGE analysis, these two isolates showed 95% similarity (two different bands).

Four pairs of isolates belonging to PFGE-E subtypes, CARB102-E1/CARB183-E16 (group a), CARB130-E12/CARB096-E13 (group b), CARB044-E17/CARB139-E18 (group c), and CARB007-E1/CARB009-E1 (group d), showed no allele differences when analyzed by cgMLST (Figure 1A). When using wgMLST only, isolates from group c had zero allele differences (Figure 1B), whereas isolates from group a showed one allele difference, isolates from group b showed four allele differences, and isolates from group d were genetically unrelated (Figure 1B).

### Analysis of Isolates Using the BIGSdb-Kp cgMLST Scheme

We obtained identical seven-gene MLST STs for all isolates when using the BIGSdb-Kp database to those previously determined using other WGS analysis tools, confirming the high reproducibility of the seven-gene MLST scheme from genomic assemblies. All ST405 isolates had ≤2 allelic mismatches (mostly 0 allelic mismatches) (Table 2; figure in Supplementary Figure S1). The three ST101 isolates presented between 2 and 5 allelic mismatches, whereas ST14, ST17, and ST1233 isolates were genetically unrelated with more than 480 allelic mismatches (figure in Supplementary Figure S1).

We also calculated the genetic distance for this scheme; all paired isolates had a genetic distance below 0.0032, except for CARB115-A1 and CARB058-A3, which had a genetic distance of 0.0080 (five allelic mismatches) (Table 2).

### Core-Genome SNP Analysis

Isolate CARB007-E1 – the oldest isolate – was used as a reference genome for the cgSNP analysis of ST405 isolates (*n* = 31). The average of core genome alignment was 88.7%. Among ST405 isolates, cgSNP distance ranged between 16 and 61 core SNPs (Supplementary Figure S2 and Supplementary Data Sheet S2). The four groups of two isolates that had identical allelic profiles, as analyzed by cgMLST (Figure 1A), showed 6, 29, 6, and 17 SNPs, respectively, for groups a, b, c, and d. The average of core genome alignment for ST101 isolates was 85.4%. ST101 isolates CARB115-A1 and CARB077-A2 showed 2,427 and 2,673 core SNPs, respectively, compared with isolate CARB058-A3, which was used as reference (Supplementary Figure S2 and Supplementary Data Sheet S2), whereas the number of core SNPs between CARB115-A1 and CARB77-A2 was 54 (Supplementary Figure S2 and Supplementary Data Sheet S2).

### Antimicrobial Resistance Genes From WGS Data Compared to PCR and Phenotypic Results

The ResFinder tool from the Center for Genomic Epidemiology detected the presence of multiple resistance genes implicated in β-lactams, aminoglycosides, and quinolone resistance as follows. All isolates were positive for *bla*_OXA–__48_, except CARB077, and for one of the following *bla*_SHV_ genes: *bla*_SHV–__1_ (*n* = 4), *bla*_SHV–__11_ (*n* = 4), *bla*_SHV–__42_ (*n* = 1), and *bla*_SHV–__76_ (*n* = 31) (Table 1). In addition, 78.4% (29/37) of the isolates contained *bla*_CTX–M–__15_; 56.4% (22/37), *bla*_OXA–__1_; and/or 81% (30/37), *bla*_TEM–__1_ genes (Table 4). We observed some discrepancies with results previously obtained by PCR and Sanger sequencing (Argente et al., 2019). In three isolates (CARB009, CARB050, and CARB056), the *bla*_OXA–__1_ gene was detected by PCR but not by WGS, whereas in two isolates (CARB039 and CARB044), the *bla*_CTX–M–__15_ gene was not detected by PCR but detected by WGS. In the latter case, the phenotypes were in concordance with the PCR results; the isolates were susceptible to third-generation cephalosporins. Finally, CARB184 isolate was positive for the *bla*_CTX–M–__15_ gene by PCR (in concordance with its antimicrobial susceptibility testing results), but this gene was not detected by WGS.

**TABLE 4:** Cluster types (CTs) and resistance genes found in the 37 carbapenemase-producing *K. pneumoniae* (except one isolate, by ResFinder, all were positive for *bla*_OXA–__48_, and for one of the following genes: *bla*_SHV–__1_, *bla*_SHV–__11_, *bla*_SHV–__42_, and *bla*_SHV–__76_).

**ST**	**CT**	**n**	**PFGE profiles**	**Isolate**	**Beta-lactamases**			**Aminoglycosides-modifying enzymes**	**Others**	**Resistome**
101	1149	1	A1	CARB115	*bla*_OXA–__1_ (100)	*bla*_TEM–__1__B_ (100)	*bla*_CTX–M–__15_ (100)	*str*A (99,88)/*str*B (99,88); *aac*(3)-IId (99,88); *aac(6′)-*Ib-cr (100)	*sul*2 (100); *cat*B3 (100); *tet*(D) (100)	
	1152	1	A2	CARB077	*bla*_OXA–__1_ (100)	*bla*_TEM–__1__B_ (100)	*bla*_CTX–M–__15_ (100)	*str*A (99,88)/*str*B (99,88); *aac*(3)-IId (99,88); *aac*(6*′*)-Ib-cr (100)	*sul*2 (100); *cat*B3 (100); *tet*(D) (100)	
	1154	1	A3	CARB058	*bla*_OXA–__1_ (100)	*bla*_TEM–__1__B_ (100)	*bla*_CTX–M–__15_ (100)	*str*A (99,88)/*str*B (99,88); *aac*(3)-IIa (99,77); *aac*(6*′*)-Ib-cr (100); *aph*(3*′*)-Ia (100)	*sul*2 (100); *cat*B3 (100); *tet*(D) (100)	

405	185	7	E4; E5; E7; E14; E17; E18 (2)	CARB^*a*^	*bla*_OXA–__1_ (100)	*bla*_TEM–__1__B_ (100)	*bla*_CTX–M–__15_ (100)	*str*A (100)/*str*B (100); *aac*(3)-IIa (99,77); *aac*(6*′*)-Ib-cr (100)	*sul*2 (100); *tet(A)* (100); *catB*3 (100)	I
	185	3	E1	CARB^*b*^		*bla*_TEM–__1__B_ (100)	*bla*_CTX–M–__15_ (100)	*str*A (100)/*str*B (100); *aac*(3)-IIa (99,77)	*sul*2 (100)	II
	185	1	E1	CARB015	*bla*_OXA–__1_ (100)	*bla*_TEM–__1__B_ (100)	*bla*_CTX–M–__15_ (100)	*str*A (100)/*str*B (100); *aac*(3)-IIa (99,77); *aac(6′)-*Ib-cr (100)	*sul*2 (100); *tet(A)* (100)	III
	185	1	E15	CARB026	*bla*_OXA–__1_ (100)	*bla*_TEM–__1__B_ (100)	*bla*_CTX–M–__15_ (100)	*str*A (100)/*str*B (100); *aac(6′)-*Ib-cr (100);	*sul*2 (100); *tet(A)* (100); *cat*B3 (100)	V
	185	1	E1	CARB011		*bla*_TEM–__1__B_ (100)	*bla*_CTX–M–__15_ (100)	*str*A (100)/*str*B (100)	*sul*2 (100)	VI

	1150	8	E2; E5 (2); E6; E8; E12 (2); E16	CARB^*c*^	*bla*_OXA–__1_ (100)	*bla*_TEM–__1__B_ (100)	*bla*_CTX–M–__15_ (100)	*str*A (100)/*str*B (100); *aac*(3)-IIa (99,77); *aac*(6*′*)-Ib-cr (100)	*sul*2 (100); *tet(A)* (100); *catB*3 (100)	I
	1150	1	E5	CARB056		*bla*_TEM–__1__B_ (100)	*bla*_CTX–M–__15_ (100)	*str*A (100)/*str*B (100); *aac*(3)-IIa (99,77)	*sul*2 (100)	II
	1150	1	E1	CARB037	*bla*_OXA–__1_ (100)	*bla*_TEM–__1__B_ (100)	*bla*_CTX–M–__15_ (100)	*str*A (100)/*str*B (100); *aac*(3)-IIa (99,77); *aac(6′)-*Ib-cr (100)	*sul*2 (100); *tet(A)* (100)	III
	1150	2	E1 (1); E6 (1)	CARB125/116	*bla*_OXA–__1_ (100)			*aac(6′)-*Ib-cr (100)	*tet(A)* (100): *cat*B3 (100)	IV
	1150	1	E10	CARB039	*bla*_OXA–__1_ (100)	*bla*_TEM–__1__B_ (100)	*bla*_CTX–M–__15_ (100)	*str*A (100)/*str*B (100); *aac(6′)-*Ib-cr (100);	*sul*2 (100); *tet(A)* (100); *cat*B3 (100)	V
	1150	1	E1	CARB102	*bla*_OXA–__1_ (100)			*aac(6′)-*Ib-cr (100)	*cat*B3 (100)	VII
	1150	1	E13	CARB96	*bla*_OXA–__1_ (100)	*bla*_TEM–__1__B_ (100)	*bla*_CTX–M–__15_ (100)	*str*A (100)/*str*B (100); *aac*(3)-IIa (99,77); *aac(6′)-*Ib-cr (100)	*sul*2 (100); *cat*B3 (100)	VIII
	1150	1	E3	CARB042	*bla*_OXA–__1_ (100)			*aac*(3)-IIa (99,77); *aac(6′)-*Ib-cr (100)	*tet(A)* (100); *cat*B3 (100)	IX
	1150	1	E11	CARB184	*bla*_OXA–__1_ (100)	*bla*_TEM–__1__B_ (100)		*aac*(3)-IIa (99,77); *aac(6′)-*Ib-cr (100)	*tet(A)* (100): *cat*B3 (100)	X

	1153	1	E9	CARB123	*bla*_OXA–__1_ (100)	*bla*_TEM–__1__B_ (100)	*bla*_CTX–M–__15_ (100)	*str*A (100)/*str*B (100); *aac*(3)-IIa (99,77); *aac*(6*′*)-Ib-cr (100)	*sul*2 (100); *tet(A)* (100); *catB*3 (100)	I

14	1151	1	D	CARB117						

17	1155	1	B	CARB098	*bla*_OXA–__1_ (100)					

1233	952	1	C	CARB122						

**In parenthesis, the percentage of homology is indicated with the following genes: bla_*OXA–*__1_ (J02967), bla_*TEM–*__1__*B*_ (JF910132), bla_*CTX–M–*__15_ (DQ302097), aph(3′)-Ia (V00359), strA (AF321551), strB (M96392), aac(3)-IId (X51534), aac(6′)-Ib-cr (DQ303918), sul2 (CQ421466), catB3 (AJ009818), tet(A) (AJ517790), and tet(D) (AF467077).All strains showed dfrA14 (99,59) (DQ388123) and oqxA (99,66)/oqxB (98,61) (EU370913) genes. All ST405 strains had qnrB66 (99,07) (KC580655) and fosA (98,81) (NZ_ACWO01000079), and the A3 strain carried qnrB1 (100) (EF682133).CARB^*a*^: E4 = CARB106, E5 = CARB022, E7 = CARB038, E14 = CARB010, E17 = CARB044, E18 = CARB040 and CARB139.CARB^*b*^: CARB007, CARB009, CARB050.CARB^*c*^: E2 = CARB020, E5 = CARB128 and CARB182, E6 = CARB065, E8 = CARB100, E12 = CARB112 and CARB130, E16 = CARB183.ST, sequence type; PFGE, pulsed-field gel electrophoresis.**

We observed five acquired genes related to aminoglycoside resistance: *str*A/*str*B (78.4%; 29/37) and *aph*(3′)-Ia (2.7%; 1/37) (streptomycin resistance), *aac*(6′)-Ib-cr (78.4%; 29/37) (kanamycin, tobramycin, amikacin, netilmicin, and fluoroquinolone resistance), *aac*(3)-IIa (70.3%; 26/37), and *aac*(3)-IId (5.4%; 2/37) (kanamycin, gentamycin, tobramycin, and netilmicin resistance).

We compared the nucleotide sequences of the *gyr*A, *gyr*B, *par*C, and *par*E genes and their amino acid sequences, of the entire isolate collection with those of the ATCC reference strain 13883, a quinolone-susceptible strain (Brisse et al., 1999). Of the 37 isolates, 29 had a ciprofloxacin minimal inhibitory concentration ≥2 mg/L, but mutations in *gyr*A and *par*C were only found in 5 isolates. The ST101 isolates CARB058, CARB077, and CARB115 had non-silent mutations in the *gyrA* gene (Ser83Tyr, Asp87Gly, or Asp87Ala) and the *parC* gene (Ser80Ile, Asn304Ser). The ST405 isolate CARB039 had a non-silent mutation in the *gyrA* gene (Ser83Ala). In addition, one ST17 isolate (CARB098) showed two new mutations in the *gyr*A gene (Ala863Val, Thr868Ile); however, the ciprofloxacin minimal inhibitory concentration for this isolate was 1 mg/L. In addition, *qnr*B66 (81.6%), *qnr*B1 (27%), and *aac*(6′)-Ib-cr genes (78.4%) were present, which could explain a certain level of quinolone resistance (Table 4). However, seven isolates carrying the *qnr*B gene (CARB007, CARB011, CARB037, CARB044, CARB096, CARB128, CARB139) and five isolates carrying the *aac*(6′)-Ib-cr gene (CARB037, CARB044, CARB096, CARB128, CARB139) were susceptible to ciprofloxacin and nalidixic acid. *Oqx*A/B genes, encoding an efflux pump, were present in all isolates. Nevertheless, this mechanism is associated with a low level of resistance to several antimicrobials, such as conferring low to intermediated resistance to quinoxalines, quinolones, tigecycline, nitrofurantoin, several detergents, and disinfectants (benzalkonium chloride, triclosan, and sodium dodecyl sulfate).

Other resistance genes identified by WGS were *sul*2 (78.4%) and *dfr*A14 (91.9%) genes (co-trimoxazole resistance), *tet*(A) (64.8%) and *tet*(D) (8.1%) genes (tetracycline resistance), and *cat*B3 (72.9%) (chloramphenicol resistance) and *fos*A (83.8%) (fosfomycin resistance).

We defined different resistance profiles (resistomes) based on the combination of acquired ARGs (Table 4). Resistome I included 14 ARGs, whereas resistomes XVI to XVIII only included 1 ARG each. We observed three different resistomes (XIII to XV) in ST101 isolates, in concordance with CTs (cgMLST) and PFGE profiles; whereas in ST405 isolates, we observed 12 resistomes (I–XII); the two most predominant ones (I and II) were shared between CT185, CT1150, and CT1153.

ST405 isolates of two of the three groups that showed no allele differences by cgMLST analysis and that had different PFGE profiles displayed a different resistance pattern. Isolates from group a had resistance patterns I/VII, and isolates from group b had resistance patterns I/III, with the difference being the presence of *tet*A gene (Table 4). In contrast, isolates from group c that had the same resistome (I/I) and the same CT were identified by wgMLST as having identical allelic profiles.

### Plasmid Analysis

Plasmid analysis revealed that all but two isolates (CARB039-E10 and CARB077-A2) were positive for a plasmid of the incompatibility group (Inc) L. All isolates also carried plasmids of Inc FIB, and all isolates except those of PFGE profiles B (ST17), C (ST1233), and D (ST14) also carried a plasmid of the Inc FIIk (Supplementary Table S2). In addition, two isolates (ST101; A1 and A2) were positive for an Inc R plasmid, and two (ST405; E4 and E5) had an Inc HI1B plasmid. Inc FIB, FII, and HI1B plasmids were not previously amplified by PCR-based replicon typing (PBRT) (Argente et al., 2019). However, Inc ColE and FIA plasmids, previously detected by PBRT, were not detected by WGS analysis since they are not included in the PlasmidFinder 1.3 tool (Supplementary Table S2). In addition, we found plasmid FIB homology with three previously described plasmids of the same incompatibility group [FIB (PKPHS1) (95.54%), FIB (Mar) (99.77%), FIB (PKPHS1) (96.01%)].

**FIGURE 2:**
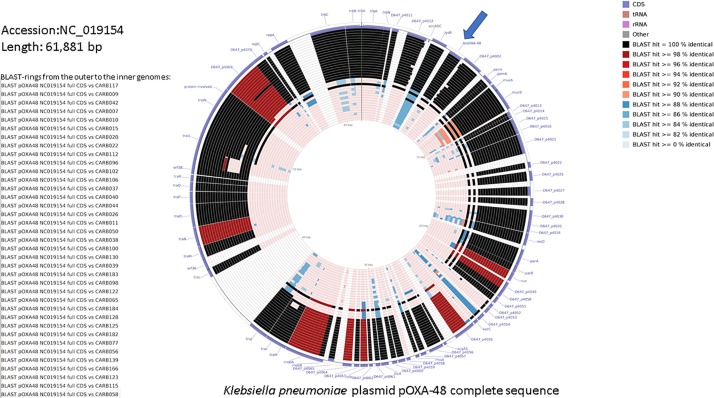
DNA comparison of the reference *K. pneumoniae* plasmid pOXA-48 (NC_019154.1) with assembled genomes using the CGView Comparison Tool (Grant et al., 2012). The legend on the left lists the 37 genome sequences used for the comparative BLAST-based analysis (genome names from the top to the bottom, and corresponding BLAST rings from the outer to the inner ones). The two outermost rings represent the forward and reverse coding strands of the plasmid reference genome. The color code for the coding strand features is highlighted in the legend on the bottom right. A separate BLAST ring is drawn for each comparison genome toward the inner part of the figure, and region of sequence similarity between the reference sequence and any part of the comparison sequence is shown in gradient color. *bla*_OXA–__48_ gene is indicated with an arrow.

Figure 2 shows a similarity of 84–100% between annotated coding sequences of the plasmid pOXA-48 reference genome and genome assemblies of OXA-48-producing *K. pneumoniae* isolates from this study. Plasmid L genetic rearrangements, such as deletions, are shown in Supplementary Figure S3 and did not show correlation with the cgMLST tree.

### Nucleotide Sequence Accession Numbers

Raw reads used in this study have been deposited to the European Nucleotide Archive of the European Bioinformatics Institute under the project accession number PRJEB27508.

## Discussion

WGS has a higher discriminatory power than do conventional typing methods and allows for a more precise outbreak analyses. This is especially important when analyzing the emergence and evolution of antimicrobial resistance, such as the threat of a worldwide spread of carbapenem resistance.

Previous studies described the molecular epidemiology of carbapenemase- and extended-spectrum beta-lactamase-producing *K. pneumoniae* isolates by WGS using a gene-by-gene comparison and/or SNP analysis (Snitkin et al., 2012; Lee et al., 2014; Marsh et al., 2015; Mathers et al., 2015; Onori et al., 2015; Zhou et al., 2015; Kluytmans-van den Bergh et al., 2016; Pérez-Vázquez et al., 2016; Zhou et al., 2016; Taboada et al., 2017). Here we combined both approaches, describing for the first time the cgMLST scheme included in SeqSphere+ Ridom software. In addition, we compared the results with those obtained with classical typing methods. For this purpose, we used a collection of OXA-48-producing *K. pneumoniae* isolates related to an increase in carbapenem resistance (ertapenem and imipenem) in Catalonia, which has been observed since 2012. The ST405 isolates were the most prevalent (77.6%) (Argente et al., 2019) and represented a predominant clone in Spain (Oteo et al., 2014). Previous bacterial-typing analysis of this collection by PFGE and MLST methods showed close genetic relatedness between all ST405 isolates, despite the fact that no clinical epidemiological relationships were established among patients.

First, we performed cgMLST and wgMLST using the BIGSdb-Kp and Ridom SeqSphere+ software tools, using a gene-by-gene approach. This reduced the analysis to coding regions but benefited from a stable nomenclature, which ensures interlaboratory reproducibility (Zhou et al., 2017). Second, we analyzed genome assemblies using the previously described Institut Pasteur cgMLST scheme (Bialek-Davenet et al., 2014), which already demonstrated its ability to subtype isolates with the same MLST denominations, and showed that MLST classification into traditional clonal complexes can merge members of distinct clonal groups (Bialek-Davenet et al., 2014). Last, we performed a cgSNP analysis of isolates of the same ST to investigate whether the isolates that did not show any allele differences by cgMLST were truly identical.

We observed two major clusters when using the cgMLST SeqSphere+ analysis. Cluster 1 included all isolates of the ST405/PFGE-E clone and comprised CTs 185, 1150, and 1153. Within cluster 1, three groups of two isolates each, each with different PFGE pattern subtypes E1/E16, E13/E12, and E17/E18 (groups a to c), showed no single allele difference using cgMLST and had a genetic distance below the threshold of 0.0035, confirming a close genetic relatedness (Kluytmans-van den Bergh et al., 2016). The PFGE and the cgMLST/wgMLST patterns were not the same, which may be explained by the fact that these two subtyping methods examine and distinguish different types of genetic mutation events (Zhou et al., 2017). Indeed, isolates could have mutations in the genome that do not affect restriction sites, and therefore, different cgMLST/wgMLST allelic profiles could have the same PFGE pattern (Tenover et al., 1995). Furthermore, PFGE diversity could be reflecting mostly changes in the accessory genome, for example, gain or loss of mobile genetic elements or recombination events, rather than true genealogies (Zhou et al., 2013).

In this study, ST405 isolates with identical cgMLST allelic profiles (SeqSphere+) showed between 6 and 17 (groups a, c, d) and 29 (group b) cgSNPs. These results are within the range of 1–77 SNPs that was previously described for ST405 isolates as sporadic (Onori et al., 2015). Other studies reported comparable results of cgMLST and SNP-based phylogeny in KPC-producing *K. pneumoniae* (Lee et al., 2014; Taboada et al., 2017). We observed 50 times more core SNPs between isolates CARB058 and CARB115 (both PFGE-A and ST101) than between isolates CARB058 and CARB077. These results were in concordance with cgMLST and wgMLST results, demonstrating again the higher discriminatory power of these two WGS-based approaches compared to PFGE and MLST.

One of the main challenges with the WGS-based typing methods (cgMLST, wgMLST, and cgSNP analysis) is to define cut-off values that can be used to identify a single-clone outbreak. Previous studies focusing on a gene-by-gene approach have used different cgMLST schemes (Lee et al., 2014; Treangen et al., 2014; Taboada et al., 2017; Zhou et al., 2017). Moreover, some authors have developed bioinformatic tools for an interactive identification of the most appropriate scheme (Silva et al., 2018). Taken together, this suggests that a definition of a unique threshold to define clonal relatedness is questionable. A previous study on *K. pneumoniae* established a maximum of 10 allele differences to cluster outbreak isolates and to separate unrelated out-group isolates when using a cgMLST scheme of 1,143 genes (Zhou et al., 2017). In this work, we applied two different cgMLST schemes, and both easily identified isolates with close genetic relatedness. However, the number of allele differences/allelic mismatches varied from 0 to 2 when using a 694-gene cgMLST scheme, and from 0 to 14 when using a 2,365-gene cgMLST scheme (Table 2), which indeed suggests that a different cluster alert distance should be applied depending on the cgMLST scheme. No threshold has been defined when using cgMLST from BIGSdb-Kp, but clearly, genetically related isolates are nearly identical (≤2 mismatches) when using this scheme. The analysis using the scgMLST BIGSdb-Kp scheme therefore provided complementary information that may be easier to interpret given the lower variation observed using this scheme.

We used two different thresholds in the gene-by-gene approach, a cluster alert distance of 15 from SeqSphere+ software (see text footnote 2) and a genetic distance of 0.0035 for cgMLST analysis described by Kluytmans-van den Bergh et al. (2016). Both proved to be useful for the description of genetic relatedness. In addition, the latter could be adopted independently of the cgMLST scheme used, which would also facilitate the interpretation of results, since it considers the proportion of shared genes by two isolates that are being compared. CARB100/CARB123 and CARB077/CARB115 isolates, with fewer than 14 allele differences (cluster alert distance 15), had a genetic distance above the threshold, which would have been interpreted as there being no genetic relatedness. However, when using the cgMLST BIGSdb-Kp scheme, the genetic distance was 0.0032 (2 allelic mismatches), indicating instead genetic relatedness when applying the same threshold. This illustrates that thresholds must be used with caution and cannot *per se* provide a definitive answer as to the genetic relatedness.

A threshold assessment is even more difficult for an SNP analysis; most authors analyzing *K. pneumoniae* isolates described the number of different SNPs detected, but no clear cut-off value has been assigned (Snitkin et al., 2012; Marsh et al., 2015; Mathers et al., 2015; Onori et al., 2015; Zhou et al., 2015; Pérez-Vázquez et al., 2016). Moreover, the number of SNPs detected depends on the reference genome used for the analysis and on the degree to which artifactual SNPs, due to paralogous sequences, for example, are filtered out during the SNP analysis. Some studies investigated the mutation rate of different species in order to define a threshold for the number of SNPs between related isolates (Köser et al., 2012; Zhou et al., 2013; Ruppitsch et al., 2015; Moura et al., 2017; Silva et al., 2018). Pérez-Vázquez et al. (2016) described an accumulation of approximately 11 SNPs per genome per year for OXA-48-*K. pneumoniae* isolates of ST405. Public Health England has published a reproducible and scalable pathogen population SNP-based analysis, including an “SNP address” nomenclature (Okoro et al., 2012).

The advantages of WGS-based typing are undeniable, however, “unique” or “one-fits-all” thresholds, by means of either gene-by-gene or SNP analysis, seem to be difficult to establish; it may be not only species-specific but also population-specific, which requires the careful and flexible application of proposed thresholds as previously discussed (Dallman et al., 2017). Nevertheless, efforts within the scientific community and public health authorities to unify the parameters and values used to define clonality would facilitate a common practice in data interpretation. This could be achieved by adopting a threshold range to be interpreted in combination with clinical epidemiological data and species population characteristics.

Regarding antimicrobial resistance mechanisms and possible vectors for the dissemination of those, we described 18 ARGs and the presence of up to four plasmids as possible disseminators of these. We established that quinolone resistance was not determined by mutations in topoisomerases as expected, but was probably due to the interplay between different mechanisms, i.e., plasmid factor QnrB66, the aminoglycoside-modifying enzyme AAC(6′)-Ib-cr, and the efflux pump OqxAB. The antibiotic resistance genotype profiles obtained by classical PCR (Argente et al., 2019) were mostly congruent with WGS results. Detection of genes by PCR, but not by WGS, could be explained by the loss of the plasmid harboring the corresponding gene or by incorrect assembly or low coverage of the respective gene (Leopold et al., 2014). Multiple factors could cause a negative PCR result, including inadvertently contamination of PCR tubes with glove power, which can inhibit steps during the PCR process (Schürch et al., 2018).

Our findings confirmed the conserved IncL plasmid as the major vector for the dissemination of OXA-48 carbapenemase. However, genetic rearrangements, such as deletions and point mutations, have also been observed within our 1-year isolate collection, reflecting genome plasticity and dynamics of horizontal gene transfer (Lomas et al., 1992). In addition, WGS allows us to identify the degree of homology between plasmid incompatibility groups, whereas by classical PBRT analysis, we only showed the presence or absence of the replicon genes. Nevertheless, the rapid advance in sequencing techniques will be decisive to describe plasmid features and sequences in accurate detail.

## Conclusion

In conclusion, we observed an overall correlation between cgMLST and wgMLST, and cgSNP-analysis results to determine similarity of OXA-48-producing *K. pneumoniae* clinical isolates. CgMLST produced high-resolution allelic profiles and different cgMLST schemes provided complementary results that allowed for a better interpretation of results. Furthermore, cgSNP-based typing was useful to resolve and better discriminate OXA-48-producing *K*. *pneumoniae* clusters within the same ST. Differences between classical and WGS-based typing methods highlight the different molecular principle of these techniques, and we emphasize the importance to include clinical epidemiological data to elucidate clonal spread.

## Data Availability Statement

The datasets generated for this study can be found in the ENA.

## Author Contributions

EM, JR, and SG-C: conception and design. EM, SB, and SG-C: acquisition of data. EM and SG-C: laboratory analysis. EM, MC, VP, and SG-C: data analysis. EM, JR, MC, DH, SB, VP, and SG-C: data interpretation. EM and SG-C: writing of the draft manuscript. EM, JR, MC, DH, SB, VP, FN, AF, and SG-C: review of the manuscript.

## Conflict of Interest

JR consults for IDbyDNA.

The remaining authors declare that the research was conducted in the absence of any commercial or financial relationships that could be construed as a potential conflict of interest.

## Footnotes

1 https://bigsdb.pasteur.fr/

2 https://www.ridom.de/u/Core_Genome_MLST_Complex_Type.html

3 https://bigsdb.pasteur.fr/klebsiella

## Supplementary Material

FIGURE S1 Minimum spanning tree (BioNumerics v6,7,3, Applied-Maths, Belgium) based on BIGSdb-Kp scgMLST allelic profile comparisons. Each circle represents a single profile. The number of allelic mismatches among profiles are given along links (excluding missing data in one or both profiles). The large circle corresponds to the dominant genotype.

FIGURE S2 **(A)** SNP-based tree of ST405 isolates using the Neighbour-Joining method. The genome reference is the first ST405 isolate collected (CARB007-E1). The number of SNPs compared to the reference (*) for the isolates that clustered together using a gene-by-gene approach (groups a, b, c and d) is indicated. The number of SNPs between these isolates themselves is also indicated in brackets. **(B)** SNP-based tree of ST101 isolates using the Neighbour-Joining method. The genome reference was the first ST101 isolate collected (CARB0058-A3). The number of SNPs compared to the reference (*) is indicated.

FIGURE S3 Phylogenetic tree based on cgMLST-SeqSphere+ (2,365 targets) and BLASTn comparison of *de novo* assembled genomes with *K. pneumoniae* plasmid pOXA-48 NC_019154.1 with at least 80% sequence similarity using DNAplotter (Carver et al., 2009) and visualized using Artemis Comparison Tool (ACT) (Carver et al., 2005). Information of hospital, isolation, and date of isolation is also included.

TABLE S1 Assembly quality using QUAST 4.5.

TABLE S2 PlasmidFinder v.1.3 and PBRT-PCR results.

DATA SHEET S1 CgMLST distance matrices for pairwise comparisons of 37 OXA-48 *K. pneumoniae* isolates.

DATA SHEET S2 VCF files for ST101, including SNP, indel, and structural variation calls information generated by Harvest software suuite (parsnp) (Treangen et al., 2014).
