# Supplementary material for: Core/Whole Genome Multilocus Sequence Typing and Core Genome SNP-Based Typing of OXA-48-Producing Klebsiella pneumoniae Clinical Isolates From Spain
Source: Front Microbiol. 2020 Jan 31;10:2961. doi: 10.3389/fmicb.2019.02961 (PMC7005014; doi:10.3389/fmicb.2019.02961)
Supplement: FIGURE S1 — Minimum spanning tree (BioNumerics v6,7,3, Applied-Maths, Belgium) based on BIGSdb-Kp scgMLST allelic profile comparisons. Each circle represents a single profile. The number of allelic mismatches among profiles are given along links (excluding missing data in one or both profiles). The large circle corresponds to the dominant genotype. [file Image_1.pdf]

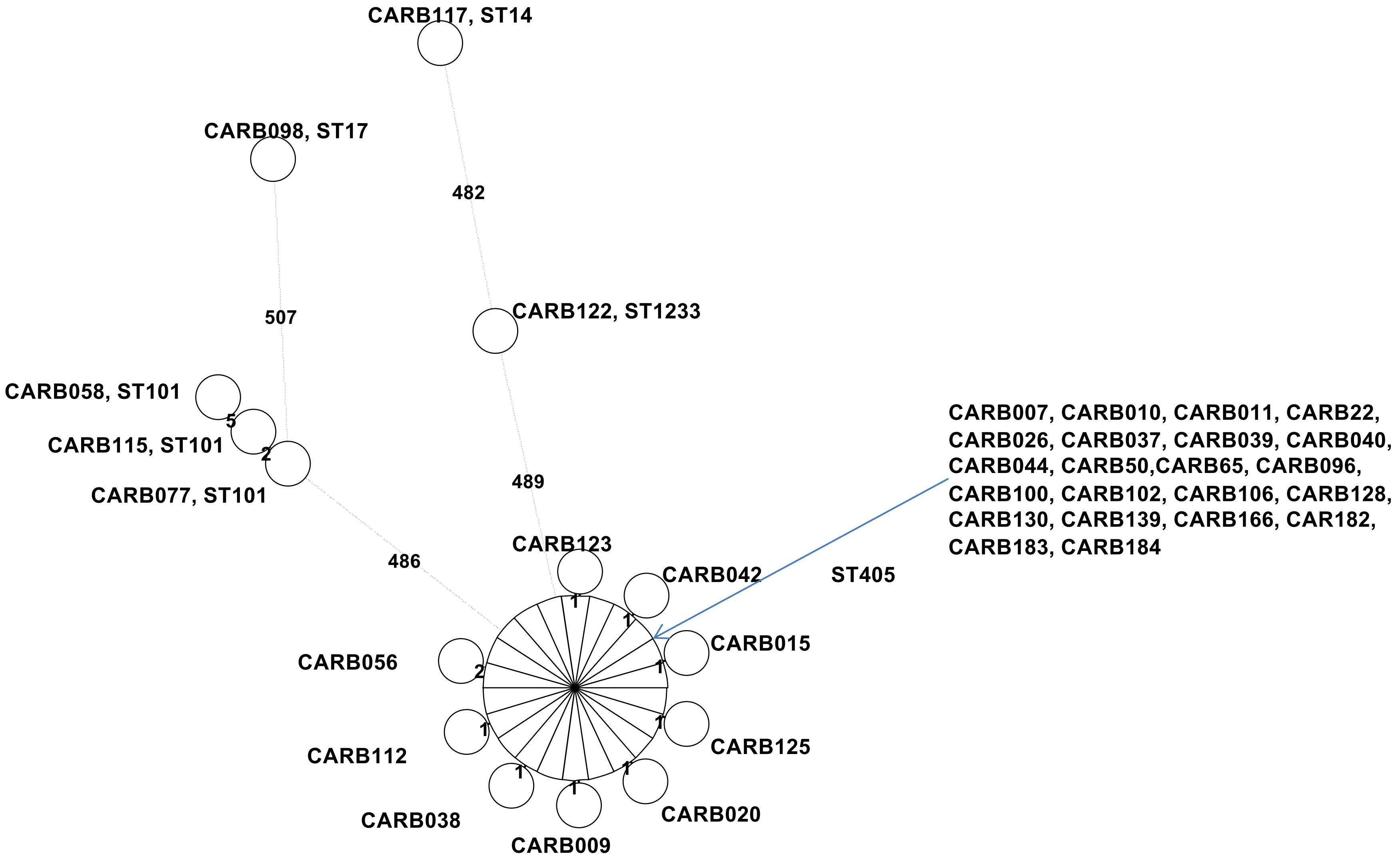

Supplementary Figure 1. Minimum spanning tree (BioNumerics v6,7,3, Applied-Maths, Belgium) based on BIGSdb-Kp scgMLST allelic profile comparisons. Each circle represents a single profile. The number of allelic mismatches among profiles are given along links (excluding missing data in one or both profiles). The large circle corresponds to the dominant genotype.
