# Supplementary material for: Core/Whole Genome Multilocus Sequence Typing and Core Genome SNP-Based Typing of OXA-48-Producing Klebsiella pneumoniae Clinical Isolates From Spain
Source: Front Microbiol. 2020 Jan 31;10:2961. doi: 10.3389/fmicb.2019.02961 (PMC7005014; doi:10.3389/fmicb.2019.02961)

Supplementary Figure 2. A) SNP-based tree of ST405 isolates using the Neighbour-Joining method. The genome reference is the first ST405 isolate collected (CARB007-E1). The number of SNPs compared to the reference (*) for the isolates that clustered together using a gene-by-gene approach (groups a, b, c and d) is indicated. The number of SNPs between these isolates themselves is also indicated in brackets. **B)** SNP-based tree of ST101 isolates using the Neighbour-Joining method. The genome reference was the first ST101 isolate collected (CARB0058-A3). The number of SNPs compared to the reference (*) is indicated.

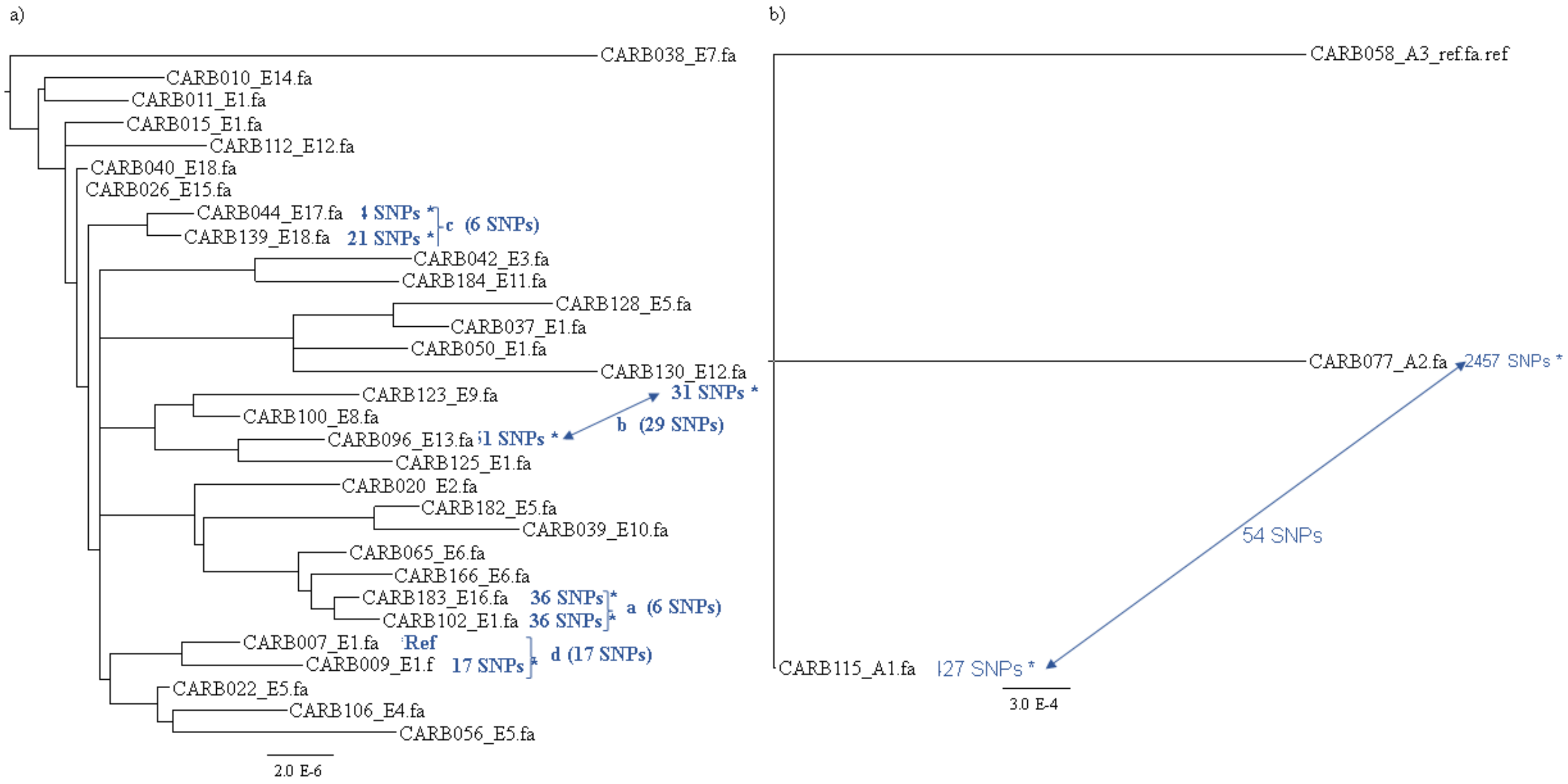

Supplement: FIGURE S2 — (A) SNP-based tree of ST405 isolates using the Neighbour-Joining method. The genome reference is the first ST405 isolate collected (CARB007-E1). The number of SNPs compared to the reference (*) for the isolates that clustered together using a gene-by-gene approach (groups a, b, c and d) is indicated. The number of SNPs between these isolates themselves is also indicated in brackets. (B) SNP-based tree of ST101 isolates using the Neighbour-Joining method. The genome reference was the first ST101 isolate collected (CARB0058-A3). The number of SNPs compared to the reference (*) is indicated. [file Image_2.pdf]
