# Supplementary material for: Core/Whole Genome Multilocus Sequence Typing and Core Genome SNP-Based Typing of OXA-48-Producing Klebsiella pneumoniae Clinical Isolates From Spain
Source: Front Microbiol. 2020 Jan 31;10:2961. doi: 10.3389/fmicb.2019.02961 (PMC7005014; doi:10.3389/fmicb.2019.02961)
Supplement: FIGURE S3 — Phylogenetic tree based on cgMLST-SeqSphere+ (2,365 targets) and BLASTn comparison of de novo assembled genomes with K. pneumoniae plasmid pOXA-48 NC_019154.1 with at least 80% sequence similarity using DNAplotter (Carver et al., 2009) and visualized using Artemis Comparison Tool (ACT) (Carver et al., 2005). Information of hospital, isolation, and date of isolation is also included. [file Image_3.pdf]

Supplementary Figure 3. Phylogenetic tree based on cgMLST-SeqSphere+ (2,365 targets) and BLASTn comparison of *de novo* assembled genomes with *K. pneumoniae* plasmid pOXA-48 NC_019154.1 with at least 80% sequence similarity using DNAplotter (Carver et al., 2009) and visualized using Artemis Comparison Tool (ACT) (Carver et al. 2005). Information of hospital, isolation, and date of isolation is also included.

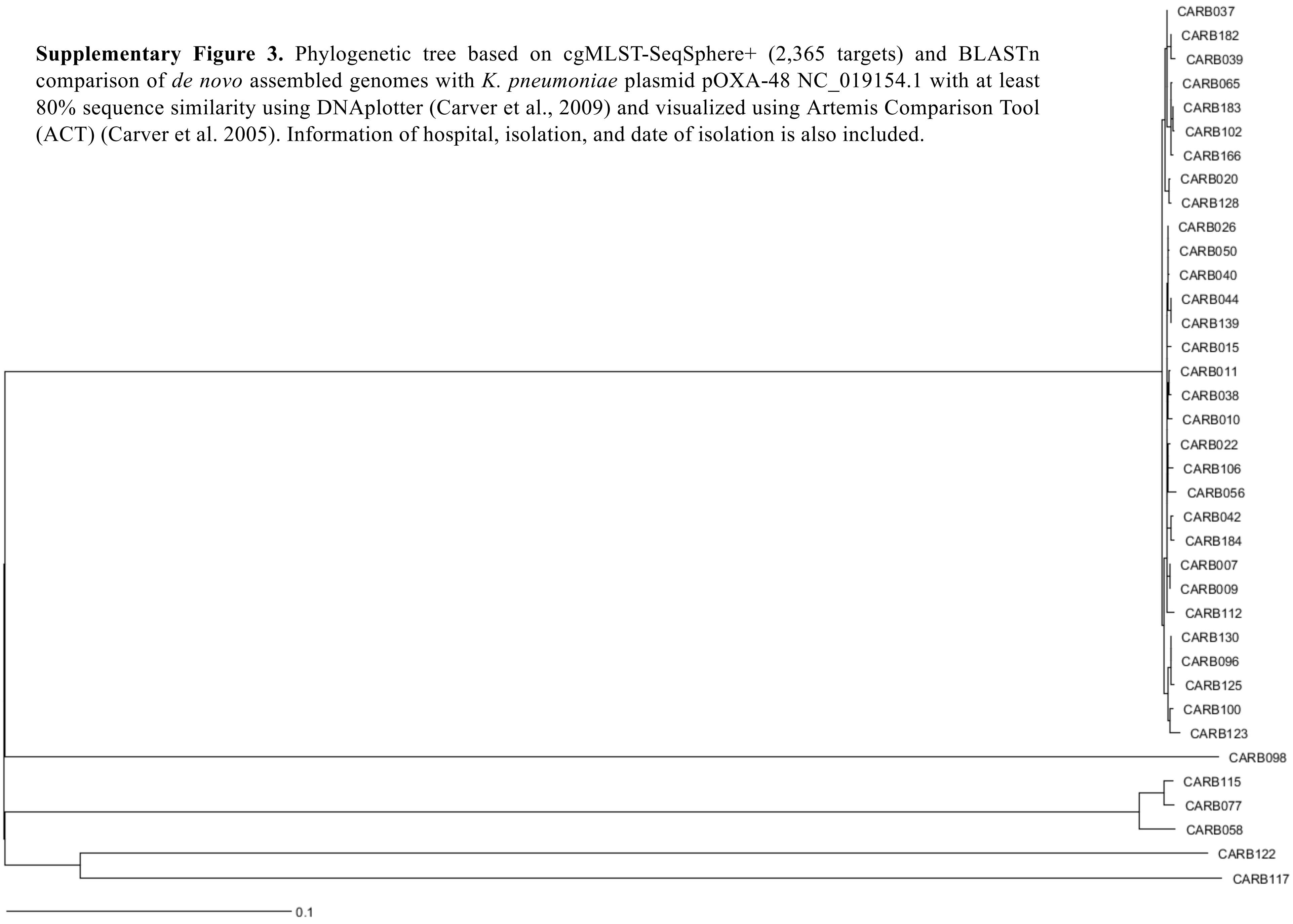

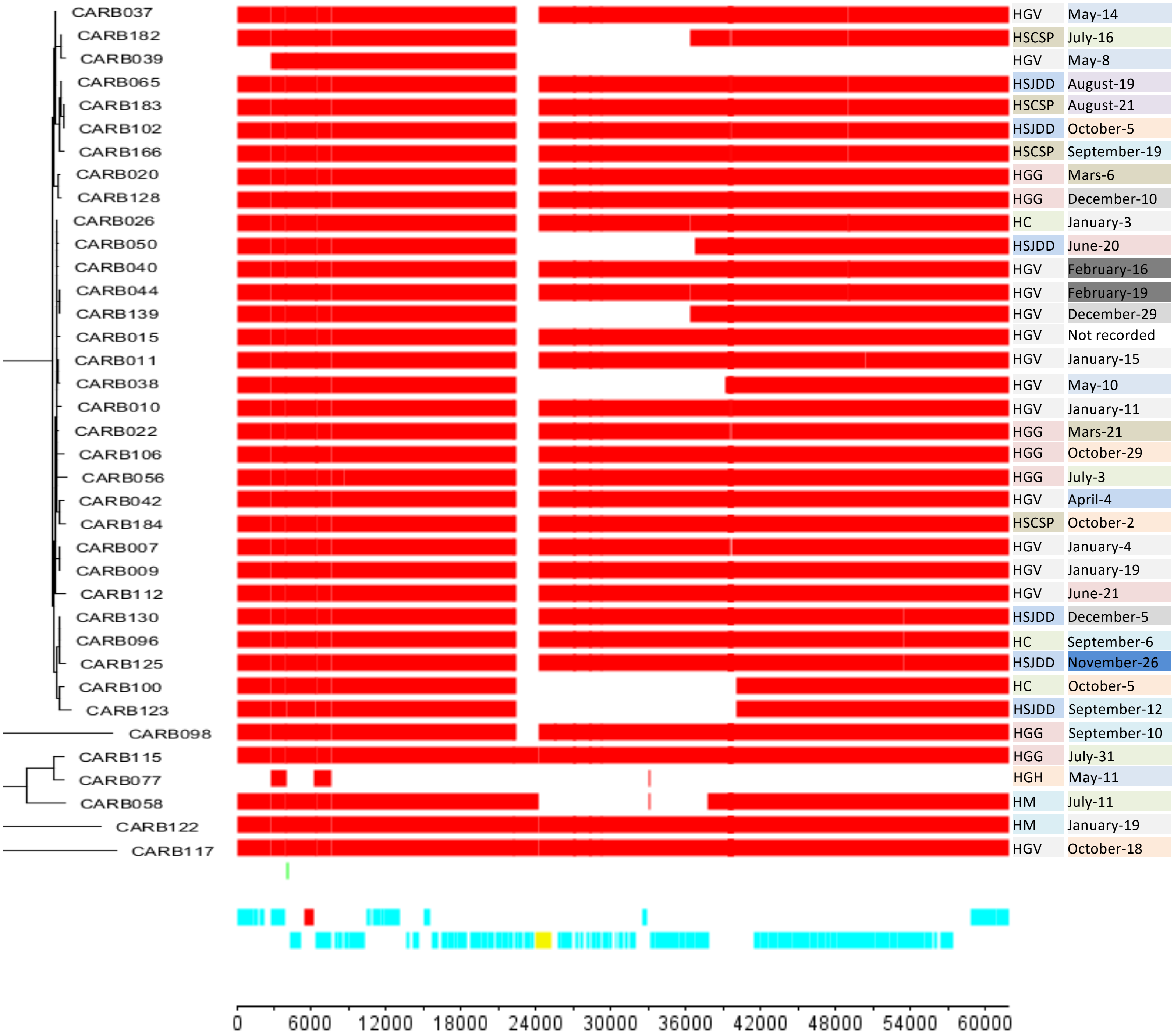

CARB037
HGV
May-14
CARB182
HSCSP
July-16
CARB039
HGV
May-8
CARB065
HSJDD
August-19
CARB183
HSCSP
August-21
CARB102
HSJDD
October-5
CARB166
HSCSP
September-19
CARB020
HGG
Mars-6
CARB128
HGG
December-10
CARB026
HC
January-3
CARB050
HSJDD
June-20
CARB040
HGV
February-16
CARB044
HGV
February-19
CARB139
HGV
December-29
CARB015
HGV
Not recorded
CARB011
HGV
January-15
CARB038
HGV
May-10
CARB010
HGV
January-11
CARB022
HGG
Mars-21
CARB106
HGG
October-29
CARB056
HGG
July-3
CARB042
HGV
April-4
CARB184
HSCSP
October-2
CARB007
HGV
January-4
CARB009
HGV
January-19
CARB112
HGV
June-21
CARB130
HSJDD
December-5
CARB096
HC
September-6
CARB125
HSJDD
November-26
CARB100
HC
October-5
CARB123
HSJDD
September-12
CARB098
HGG
September-10
CARB115
HGG
July-31
CARB077
HGH
May-11
CARB058
HM
July-11
CARB122
HM
January-19
CARB117
HGV
October-18
0
6000
12000
18000
24000
30000
36000
42000
48000
54000
60000
