# Supplementary material for: Core/Whole Genome Multilocus Sequence Typing and Core Genome SNP-Based Typing of OXA-48-Producing Klebsiella pneumoniae Clinical Isolates From Spain
Source: Front Microbiol. 2020 Jan 31;10:2961. doi: 10.3389/fmicb.2019.02961 (PMC7005014; doi:10.3389/fmicb.2019.02961)
Supplement: TABLE S1 — Assembly quality using QUAST 4.5. [file Table_1.pdf]

Report

	CARB007_trimmed	CARB009_trimmed	CARB010_trimmed	CARB011_trimmed	CARB015_trimmed	CARB020_trimmed	CARB022_trimmed	CARB026_trimmed	CARB037_trimmed
# contigs (>= 0 bp)	97	125	77	62	67	105	67	95	79
# contigs (>= 1000 bp)	91	113	70	56	61	89	61	83	70
# contigs (>= 5000 bp)	67	81	49	42	42	62	42	54	50
# contigs (>= 10000 bp)	58	65	43	38	37	53	38	48	46
# contigs (>= 25000 bp)	44	50	35	29	29	43	33	37	36
# contigs (>= 50000 bp)	35	34	26	23	23	34	26	29	27
Total length (>= 0 bp)	5687290	5681092	5802848	5622336	5688211	5574995	5884439	5774905	5691960
Total length (>= 1000 bp)	5683172	5672796	5797523	5617969	5683863	5563022	5879966	5765943	5685526
Total length (>= 5000 bp)	5621249	5588447	5744204	5577371	5639078	5495195	5830952	5695033	5632461
Total length (>= 10000 bp)	5550977	5478098	5702513	5550810	5601185	5435953	5803278	5650142	5606691
Total length (>= 25000 bp)	5295322	5237833	5558532	5406716	5465316	5271417	5717880	5481799	5408058
Total length (>= 50000 bp)	4966324	4644171	5204307	5195517	5253987	4912091	5474758	5191015	5074514
# contigs	97	125	77	62	67	105	67	95	79
Largest contig	381286	371593	559827	540642	541857	464457	541238	511073	464265
Total length	5687290	5681092	5802848	5622336	5688211	5574995	5884439	5774905	5691960
GC (%)	57.04	57.03	56.96	57.06	57.04	57.11	56.64	56.98	57.03
N50	181545	130789	297309	343335	276879	147926	276509	214728	244597
N75	83978	60244	108725	141023	172735	87029	143050	117625	124556
L50	12	13	8	7	7	12	8	9	9
L75	25	28	16	13	13	24	15	19	18
# N's per 100 kbp	0.98	3.93	0.14	1.10	0.11	2.58	6.05	0.03	4.44

	CARB038_trimmed	CARB039_trimmed	CARB040_trimmed	CARB042_trimmed	CARB044_trimmed	CARB050_trimmed	CARB056_trimmed	CARB058_trimmed	CARB065_trimmed
# contigs (>= 0 bp)	74	71	86	62	80	67	76	76	79
# contigs (>= 1000 bp)	69	64	77	58	70	62	68	71	72
# contigs (>= 5000 bp)	49	46	51	43	43	45	48	44	51
# contigs (>= 10000 bp)	42	41	45	40	40	36	44	41	43
# contigs (>= 25000 bp)	36	34	36	33	31	30	35	34	35
# contigs (>= 50000 bp)	29	26	28	27	24	22	27	21	30
Total length (>= 0 bp)	5755080	5559205	5773330	5720822	5768126	5738178	5744028	5615518	5688828
Total length (>= 1000 bp)	5751768	5554364	5766651	5717746	5760429	5734961	5737736	5611349	5683765
Total length (>= 5000 bp)	5698545	5508440	5702874	5673517	5690618	5694089	5695385	5534624	5633408
Total length (>= 10000 bp)	5648814	5473158	5655299	5651887	5668670	5633647	5666976	5512590	5582751
Total length (>= 25000 bp)	5553141	5344003	5499924	5538911	5538898	5539315	5525369	5390437	5440452
Total length (>= 50000 bp)	5303587	5034529	5215829	5310074	5292842	5242063	5234044	4967174	5255613
# contigs	74	71	86	62	80	67	76	76	79
Largest contig	394113	509693	511073	541515	541827	662917	765625	701656	765129
Total length	5755080	5559205	5773330	5720822	5768126	5738178	5744028	5615518	5688828
GC (%)	57.00	57.05	56.98	57.03	56.98	56.99	56.79	57.01	57.03
N50	275842	255308	257669	293064	277085	343269	235241	285358	215922
N75	105848	139119	117625	141017	150152	141077	114237	173932	104919
L50	9	8	8	8	7	6	7	7	9
L75	18	15	18	15	14	12	16	13	18
# N's per 100 kbp	7.65	5.95	0.07	3.43	0.94	1.43	3.31	1.76	0.11

	CARB077_trimmed	CARB096_trimmed	CARB098_trimmed	CARB100_trimmed	CARB102_trimmed	CARB106_trimmed	CARB112_trimmed	CARB115_trimmed	CARB117_trimmed	CARB122_trimmed
# contigs (>= 0 bp)	127	95	96	66	69	91	61	82	41	110
# contigs (>= 1000 bp)	114	88	91	60	64	84	56	76	39	100
# contigs (>= 5000 bp)	76	64	62	40	47	60	37	47	25	79
# contigs (>= 10000 bp)	65	58	56	33	44	56	32	43	25	69
# contigs (>= 25000 bp)	56	43	39	28	36	49	27	34	24	54
# contigs (>= 50000 bp)	37	31	27	22	30	35	20	27	21	35
Total length (>= 0 bp)	5713892	5682141	5861820	5721236	5709634	5994726	5719736	5812692	5562510	5778579
Total length (>= 1000 bp)	5703961	5677171	5857531	5717029	5705701	5989304	5716199	5808135	5560747	5771350
Total length (>= 5000 bp)	5606017	5619379	5795008	5667462	5658460	5927014	5667230	5742203	5522990	5720103
Total length (>= 10000 bp)	5529838	5577527	5753219	5615445	5640272	5896016	5630077	5713307	5522990	5649780
Total length (>= 25000 bp)	5372595	5311087	5478566	5523121	5508893	5762837	5531281	5557223	5506304	5400945
Total length (>= 50000 bp)	4731787	4874226	5051530	5292813	5299758	5269749	5275045	5336591	5404564	4691036
# contigs	127	95	96	66	69	91	61	82	41	110
Largest contig	413472	540280	600370	805897	400242	608123	541022	771146	871088	361694
Total length	5713892	5682141	5861820	5721236	5709634	5994726	5719736	5812692	5562510	5778579
GC (%)	56.64	57.03	56.72	57.03	57.01	56.50	57.07	56.54	57.16	56.83
N50	116736	185821	294951	277018	244665	156907	424158	253993	366905	135968
N75	70728	95455	95545	180705	130513	84884	217319	127354	173541	58451
L50	15	11	8	6	10	11	6	8	6	14
L75	30	22	18	12	18	24	11	16	11	29
# N's per 100 kbp	0.84	1.67	4.79	2.17	2.28	0.60	4.74	4.20	3.79	5.88

	CARB123_trimmed	CARB125_trimmed	CARB128_trimmed	CARB130_trimmed	CARB139_trimmed	CARB166_trimmed	CARB182_trimmed	CARB183_trimmed	CARB184_trimmed
# contigs (>= 0 bp)	67	104	77	91	64	62	70	97	63
# contigs (>= 1000 bp)	62	94	68	86	57	58	62	88	57
# contigs (>= 5000 bp)	44	69	50	62	40	40	41	60	40
# contigs (>= 10000 bp)	38	64	43	55	36	35	37	48	36
# contigs (>= 25000 bp)	29	54	34	44	30	28	29	37	31
# contigs (>= 50000 bp)	24	34	27	34	24	23	22	31	25
Total length (>= 0 bp)	5714901	5684936	5678653	5689126	5704259	5673515	5677943	5723083	5825104
Total length (>= 1000 bp)	5711495	5677604	5672324	5685508	5698626	5670331	5672121	5715836	5820736
Total length (>= 5000 bp)	5668606	5611971	5621902	5621574	5657680	5622642	5613932	5645471	5782264
Total length (>= 10000 bp)	5627014	5574166	5569667	5570806	5627515	5594080	5586801	5561933	5754270
Total length (>= 25000 bp)	5465130	5406603	5407992	5385371	5538630	5470575	5457771	5382810	5662147
Total length (>= 50000 bp)	5291340	4699830	5171390	4988087	5335153	5305226	5194884	5189811	5438488
# contigs	67	104	77	91	64	62	70	97	63
Largest contig	541345	442048	642127	515876	541815	766988	765046	491636	606406
Total length	5714901	5684936	5678653	5689126	5704259	5673515	5677943	5723083	5825104
GC (%)	57.03	57.04	57.06	57.03	57.03	57.04	57.05	57.01	56.89
N50	372160	127272	231528	160042	310093	297310	381142	189241	273809
N75	150152	71967	141017	88608	150152	170886	150152	117761	141186
L50	7	13	8	12	7	6	6	10	7
L75	12	27	16	24	13	12	12	20	14
# N's per 100 kbp	6.26	1.51	3.10	4.11	0.11	5.60	2.06	2.87	4.17

All statistics are based on contigs of size >= 500 bp, unless otherwise noted
(e.g., "# contigs (>= 0 bp)" and "Total length (>= 0 bp)" include all contigs).
