# Supplementary material for: Core/Whole Genome Multilocus Sequence Typing and Core Genome SNP-Based Typing of OXA-48-Producing Klebsiella pneumoniae Clinical Isolates From Spain
Source: Front Microbiol. 2020 Jan 31;10:2961. doi: 10.3389/fmicb.2019.02961 (PMC7005014; doi:10.3389/fmicb.2019.02961)
Supplement: TABLE S2 — PlasmidFinder v.1.3 and PBRT-PCR results. [file Table_2.docx]

**Supplementary Table 2.** PlasmidFinder v.1.3 and PBRT-PCR results.

| **ST** | | **PFGE** | | **PlasmidFinder** | | | | | | | **PBRT-PCR** | | |  |  |
| --- | --- | --- | --- | --- | --- | --- | --- | --- | --- | --- | --- | --- | --- | --- | --- |
| 101 | A1, A2 | | L/M (pOXA-48) (100) | | FIB (K) (98.93); FIB (pKPHS1) (95.54) |  | | R (100) | | | | L/M, R, ColE | | |  |
|  | A3 | | L/M (pOXA-48) (100) | | FIB (K) (98.93) | FII (96.83) | |  | | | | L/M, ColE | | |  |
| 17 | B | | L/M (pOXA-48) (100) | | FIB (K) (98.93); FIB (Mar) (99.77) |  | |  | | | | L/M | | |  |
| 1233 | C | | L/M (pOXA-48) (100) | | FIB (K) (98.93); FIB (Mar) (99.77) |  | |  | | | | L/M, ColE | | |  |
| 14 | D | | L/M (pOXA-48) (100) | | FIB (K) (98.93) |  | |  | | | | L/M | | |  |
| 405 | E1 (7), E2, E3, E5 (3), E6 (2), E7, E9, E12 (2), E13, E15, E18 (2) | | L/M (pOXA-48) (100) | | FIB (K) (98.93) | FII (K) (95.95) | |  | | | | L/M | | |  |
|  | E1, E8, E14, E17 | | L/M (pOXA-48) (100) | | FIB (K) (98.93) | FII (K) (95.95) | |  | | | | L/M, ColE | | |  |
|  | E4 | | L/M (pOXA-48) (100) | | FIB (Mar) (100); FIB (K) (98.93); FIB (pKPHS1) (95.36) | FII (K) (95.95) | | | HI1B (99.47) | | | | L/M | | |
|  | E5 | | L/M (pOXA-48) (100) | | FIB (Mar) (100); FIB (K) (98.93) | FII (K) (95.95) | | | HI1B (99.47) | | | | L/M | | |
|  | E10 | |  | | FIB (K) (98.93) | FII (K) (95.95) | | |  | | | | L/M | | |
|  | E11 | | L/M (pOXA-48) (100) | | FIB (K) (98.93); FIB (pKPHS1) (96.01) | FII (K) (95.95) |  | | | L/M | | | |  |  |
|  | E16 | | L/M (pOXA-48) (100) | | FIB (K) (98.93) | FII (K) (95.95) | | |  | | | | L/M, FIA, F | | |

The GenBank accession number of the cited plasmid sequences were: L/M (pOXA-48) (JN626286), FIB (K) (JN233704), FIB (pKPHS1) (CP003223), FIB (Mar) (JN420336), FII (K) (CP000648), R (DQ449578), HI1B (JN420336).
